# Can the Multidimensional Prognostic Index (MPI) be a predictive instrument for mortality in older adult liver transplant candidates?

**DOI:** 10.1007/s41999-023-00826-6

**Published:** 2023-07-18

**Authors:** Edoardo Vogliotti, Chiara Ceolin, Matteo Valenti, Jessica Vanin, Carlotta Campodall’Orto, Marta Tonon, Bruno Micael Zanforlini, Chiara Curreri, Maria Devita, Marina De Rui, Alessandra Coin, Umberto Cillo, Patrizia Burra, Paolo Angeli, Giuseppe Sergi

**Affiliations:** 1 Division of Geriatrics, Department of Medicine, University of Padua, Via Giustiniani 2, 35128 Padua, Italy; 2 Internal Medicine and Hepatology Unit, Department of Medicine, University of Padua, Padua, Italy; 3 Department of General Psychology (DPG), University of Padua, Padua, Italy; 4 Hepatobiliary Surgery and Liver Transplantation, Department of Surgery, Oncology and Gastroenterology, University of Padova, Padua, Italy; 5 Multivisceral Transplant Unit, Department of Surgery, Oncology and Gastroenterology, University of Padova, Padua, Italy; 6 Division of Geriatrics, University Hospital of Padua, Padua, Italy; 7 Present Address: Division of Geriatrics, Department of Medicine, University of Padua, Via Giustiniani 2, 35128 Padua, Italy

**Keywords:** Older adults, Liver transplantation, MPI, Liver frailty index, Mortality

## Abstract

**Purpose:**

The most recent guidelines recommend that selection of liver transplant recipient patients be guided by a multidimensional approach that includes frailty assessment. Different scales have been developed to identify frail patients and determine their prognosis, but the data on older adult candidates are still inconclusive. The aim of this study was to compare the accuracy of the Liver Frailty Index (LFI) and the Multidimensional Prognostic Index (MPI) as predictors of mortality in a cohort of older people patients being evaluated for liver transplantation.

**Methods:**

This retrospective study was conducted on 68 patients > 70 years being followed at the University Hospital of Padua in 2018. Clinical information on each patient, Model For End-Stage Liver Disease (MELD), Body Mass Index (BMI), Activities of Daily Living (ADL), Mini Nutritional Assessment (MNA), LFI, MPI, and date-of-death, were recorded. The observational period was 3 years.

**Results:**

We studied 68 individuals (25 women), with a mean age 72.21 ± 1.64 years. Twenty-five (36.2%) patients died during the observational period. ROC curve analysis showed both MPI and LFI to be good predictors of mortality (AUC 0.7, *p* = 0.007, and AUC 0.689, *p* = 0.015, respectively). MELD (HR 1.99, *p* = 0.001), BMI (HR 2.34, *p* = 0.001), and poor ADL (HR 3.34, *p* = 0.04) were risk factors for mortality in these patients, while male sex (HR 0.1, *p* = 0.01) and high MNA scores (HR 0.57, *p* = 0.01) were protective factors.

**Conclusion:**

Our study confirmed the prognostic value of MPI in older adult patients awaiting liver transplantation. In this cohort, good nutritional status and male sex were protective factors, while high MELD and BMI scores and poor functional status were risk factors.

## Key summary points

**Aim:**

The aim of this study was to compare the accuracy of the Liver Frailty Index (LFI) and the Multidimensional Prognostic Index (MPI) as predictors of mortality in a cohort of older adult patients being evaluated for liver transplantation

**Findings:**

On the 68 patients studied, ROC curve analysis showed that MPI was similar or slightly better than LFI as predictor of mortality (AUC 0.7, *p*=0.007, and AUC 0.689, *p*=0.015, respectively).

**Message:**

In older people patients listed for liver transplantation, MPI is as good a prognostic tool as LFI for predicting mortality.

## Introduction

Cirrhosis affects about 1.5 billion people worldwide, but its prevalence in the older adults population is probably underestimated due to often subtle clinical signs and symptoms [1]. All patients with end-stage liver disease should be considered for liver transplantation, a procedure that increases expectation and quality of life compared with the natural prognosis of the disease [2]. Epidemiology of end-state liver disease is partly affected by gender, probably due to social and biological differences between the two sexes [3]. If on one hand estrogens seem to slow down the progression of fibrosis in women [3], the prevalence of hepatocellular carcinoma (HCC) is higher in men. However, women appear to experience a more severe disease course with high mortality rates [3]. Regarding age, until the mid-1990s, recipients could not be over 45–50 years [4], but advances in surgical techniques and targeted immunosuppression have allowed for the removal of age as a factor in patient selection, resulting in an increase in the population that can access transplantation [5]. Consistent with the progressive aging of the population, the European Liver Transplant registry (ELTR) reports that between 2000 and 2020, the proportion of transplant patients aged > 70 increased from 0.3 to 2% [6].

The question now is how to select those patients who may benefit most from a transplant. The European Association for the Study of the Liver (EASL) guidelines recommend a multidisciplinary and multidimensional approach to selecting the recipient patient [2, 7]. In accordance with these guidelines and in light of the impact of frailty on many chronic pathologies, in recent years, assessment of patient frailty has been introduced into the pre-transplant assessment process [8]. Although the available data on older adults are inconsistent [9–11], frailty has been confirmed as an unfavorable prognostic factor in cirrhotic patients, responsible for an increased incidence of, in particular, ascites (57 vs 34%) and encephalopathy (26 vs 17%), as well as mortality [12]. The most commonly used method for assessing frailty in cirrhotic patients is the Liver Frailty Index (LFI) developed in 2019 by Lai et al., which uses physical performance-based measures. LFI has been found to be valid in estimating the mortality risk of cirrhotic patients on the transplant waiting list [13], although to date there have been few studies of older adults and these have been inhomogeneous.

Evidence from geriatric clinical practice suggests that accurate prognosis of complex patients, such as those affected by terminal hepatopathy, require multidimensional assessment. In this regard, the Multidimensional Prognostic Index (MPI) is commonly used in clinical decision making because it captures different aspects of frailty, such as nutritional and cognitive status, polypharmacy, cohabitation status, and comorbidities [14, 15]. In hospitalized older person, MPI identifies groups at risk of several outcome [16]: it has been validated as a prognostic tool for older populations in the decision-making processes of common interventions (e.g., transcatheter aortic valve implantation—TAVI) [17, 18], but so far no studies have investigated its possible role in the field of liver transplantation.

Based on these premises, the aim of this study was to compare the efficacy of LFI and MPI in estimating the risk of death in a cohort of older adult patients being evaluated for liver transplantation. A further aim was to estimate the main risk factors for death in these participants.

## Methods

### Study population

This observational retrospective study was conducted on 68 patients being followed by the Regional Center for Liver Diseases, and the Multivisceral Transplantation, Gastroenterology and Hepato-Biliary Surgery Departments of the University Hospital of Padua (Italy). During pre-transplant hospitalization, patients were also evaluated by a team of specialists from the Geriatric Unit of the same hospital. Our participants were taken in charge in 2018 and followed-up for the next 3 years. Inclusion criteria for enrollment in the study were: age > 70 years; chronic liver disease in an advanced stage (end-stage liver disease); the ability to provide informed consent for the processing of personal data, understand the tests administered, and perform physical performance tests.

The study was carried out in strict compliance with the ISHLT ethics statement. The study protocol was approved by the local Ethics Committee (Comitato Etico per la Sperimentazione Clinica di Padova, number 0014675) and complied with the guidelines of the Declaration of Helsinki. Each participant gave written informed consent to participate in the study.

### Data collection

Trained physicians gathered the following information from each participant:

*Patient* *characteristics*: Physiological, clinical, and pharmacological data were collected from each participant during a medical interview with a skilled physician. Smoking and alcohol consumption habits, social and environmental conditions, and MELD, MELD-Na, and Child–Turcotte–Pugh scores were reported. The Cumulative Illness Rating Scale (CIRS) [19] was used to assess comorbidities; Activities of Daily Living (ADL) [20] was used to determine functional autonomy; the Exton Smith Scale (ESS) [21] to determine the risk of developing pressure sores; the Mini Nutritional Assessment (MNA) [22] to determine nutritional status and the Mini-Mental State Examination (MMSE) [23] to assess cognitive performance. Finally, we calculated the Multidimensional Prognostic Index (MPI) [24] from information obtained from the SPMSQ, ESS, ADL, IADL, MNA, CIRS, the number of drugs taken by the patient, and cohabitation status to predict mortality. Body weight and height were measured with participants wearing light indoor clothing and without shoes; the body mass index (BMI) was calculated as the ratio of weight to height squared (kg/m^2^).

*Physical* *performance measures*: Grip strength was evaluated by trained medical personnel using DynEx electronic hand dynamometers (Ohio, USA). Lower extremity physical performance was assessed with the Short Physical Performance Battery (SPPB) [25].

*Frailty* *evaluations*: We used the index validated for hepatopathic patients:

- Liver Frailty Index (LFI) [13]: this measures maximum strength with the dominant hand using a dynamometer, the time in seconds the patient takes to stand up five times with arms crossed on the chest, and the time in seconds the patient is able to maintain balance in three positions (feet side by side, semi-tandem, and tandem) up to a maximum of 10 s. Patients with scores > 4.4 are considered frail.

### Statistical analysis

The characteristics of the sample compared by gender are expressed as means ± standard deviation for the continuous quantitative variables with a normal distribution, and as medians (25–75th percentiles) for those with a non-normal distribution. The normality of the distributions of the continuous quantitative variables was verified by the Shapiro–Wilk test. Categorical variables are expressed in numbers and percentages. The characteristics of the study participants were compared according to their degree of frailty using the Student’s *t* test or Chi-square test depending on the type of variable. The abilities of LFI and MPI to predict mortality were compared by receiver operating characteristic (ROC) curve analysis, and measurement of the area under the curve (AUC). Finally, the predictors of survival/mortality in the whole sample were analyzed by Cox regression, considering MELD but not CTP because of their collinearity. The statistical tests were considered significant at *p* < 0.05. All analyses were performed with IBM SPSS Statistics 25.0 (Armonk, NY: IBM Corp).

## Results

### Characteristics of the sample at baseline

Table 1 shows the characteristics of the sample at baseline by sex. Of the 68 patients studied, 25 (36.7%) were women. There were no differences in age, BMI, and social context between the sexes. A higher percentage of men than women consumed alcohol (60.5% vs. 25%, *p* = 0.01), and were active smokers (55.8% vs. 24%, *p* = 0.01) as well as smoking more cigarettes per day than their female counterparts. Regarding comorbidities, ascites, and encephalopathy were more common in females (60% vs. 25.6% and 36% vs. 11.6%, respectively), while hepatocellular carcinoma was more common in males (76.6% vs. 48%). There were no differences in BMI between people with ascites and people without ascites in both men and women (data not shown). Finally, no significant differences between the sexes in LFI and MPI were found.

**Table 1:** Characteristics of the sample at the baseline by sex

Variable	Total sample (*n* = 68)	Women (*n* = 25)	Men (*n* = 43)	*p* value
Age [years]	72.21 ± 1.64	72.12 ± 1.36	72.26 ± 1.80	0.75
BMI [Kg/m^2^]	25.06 ± 3.20	25.01 ± 3.30	25.08 ± 3.17	0.93
Social context				0.23
Living with family	63 (94%)	22 (88%)	41 (97.6%)	
Living alone	3 (4.5%)	2 (8%)	1 (2.4%)	
Recent weight loss [%]	9 (13%)	2 (8.3%)	7 (16.3%)	0.47
N drugs	5.38 ± 2.74	5.52 ± 2.60	5.30 ± 2.85	0.75
Lifestyle				
Active smoker [%]	30 (44.2%)	6 (24%)	24 (55.8%)	**0.01**
N cigarettes/day	7.82 ± 15.45	1.64 ± 3.64	11.42 ± 18.49	**0.01**
Alcohol consumption [%]	32 (47.8%)	6 (25%)	26 (60.5%)	**0.01**
Comorbidities				
CIRS-CI	2.91 ± 1.71	2.64 ± 1.65	3.07 ± 1.75	0.32
Ascites	26 (38.2%)	15 (60%)	11 (25.6%)	**0.01**
Hepatocellular carcinoma (HCC)	45 (66.2%)	12 (48%)	33 (76.6%)	**0.02**
Encephalopathy	14 (20.6%)	9 (36%)	5 (11.6%)	**0.03**
Model for End-Stage Liver Disease	14.48 ± 6.67	15.61 ± 6.06	13.88 ± 6.97	0.30
Child–Turcotte–Pugh	7.65 ± 2.33	8.29 ± 2.38	7.29 ± 2.45	0.09
Liver Frailty Index	4.03 ± 1.16	4.18 ± 0.74	3.94 ± 1.32	0.53
Multidimensional Prognostic Index	0.20 ± 0.13	0.22 ± 0.12	0.19 ± 0.75	0.48
Functional evaluation				
Total SPPB	8.30 ± 3.94	7.79 ± 3.80	8.58 ± 4.05	0.45
Max handgrip strength [Kg_f_]	26.40 ± 8.99	21.33 ± 6.29	29.13 ± 9.10	** < 0.001**
Activities of daily living	5.46 ± 1.15	5.36 ± 1.41	5.51 ± 0.98	0.60
Mini nutritional assessment	23.05 ± 4.32	22.36 ± 4.64	23.45 ± 4.13	0.33
Mini-mental state examination	26.50 ± 3.32	26.30 ± 4.22	26.51 ± 2.72	0.78

Results are expressed as means ± standard deviation or percentages (%)

*p*-values < 0.05 are identified in bold

*BMI* Body Mass Index, *CIRS-CI* Cumulative Illness Rating Scale-Comorbidity Index, *SPPB* Short physical performance battery

### Mortality

Of the patients evaluated, 34 (12.7%) were on the transplant waiting list, but only 19 were ultimately transplanted. About 23% of the patients on the list died. After 3 years, 25 (36.23%) patients had died: 10 women (41.7%) and 15 men (36.6%) (*p* = 0.79).

**Fig. 1:**
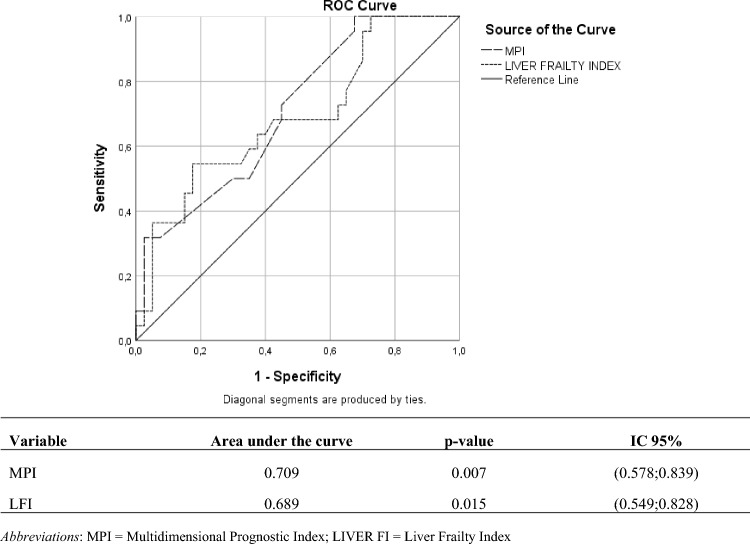
Comparison of the accuracies of the Liver Frailty Index and MPI in predicting mortality: ROC curves

The diagnostic accuracy of the LFI and MPI scales in predicting mortality was evaluated by analyzing the respective ROC curves (Fig. 1). The results showed that both had significant diagnostic accuracy (MPI: AUC = 0.709, *p* = 0.007, LFI: AUC = 0.689, *p* = 0.015).

**Fig. 2:**
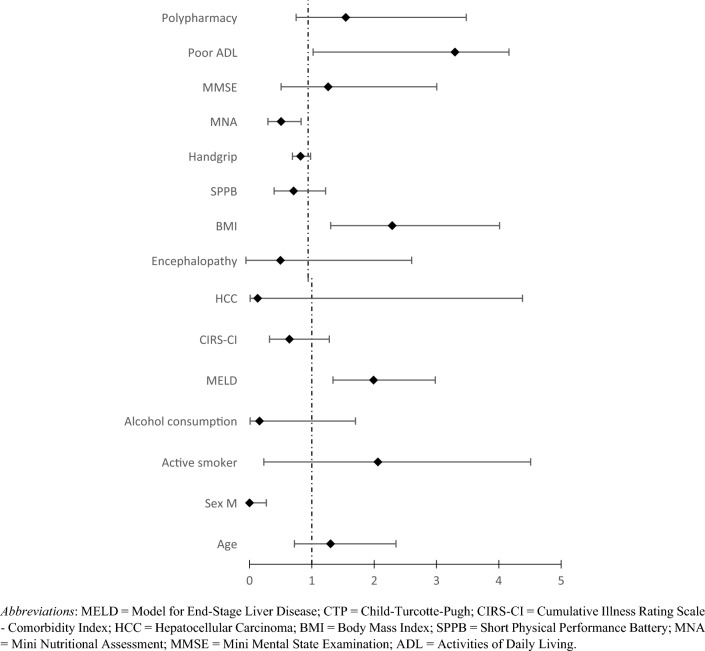
Forest plot of predictors of mortality in the sample

The Cox regression adjusted for sample characteristics (age, gender, comorbidities) is shown in Table 2 and Fig. 2. The variables significantly associated with an increased risk of death after 3 years were height MELD (HR 1.99, 95% CI 1.34–2.98, *p* < 0.001) and BMI (HR 2.34, 95% CI 1.36–4.05, *p* < 0.001), and poor ADL (HR 3.34, 95% CI 1.08–4.20, *p* = 0.04). Conversely, protective factors for mortality were male sex (HR 0.1, 95% CI 0–0.27, *p* = 0.01) and high MNA scores (HR 0.57, 95% CI 0.36–0.89, *p* = 0.01).

**Table 2:** Cox regression of covariate-adjusted mortality

Outcome	Variable	HR	*p* value	IC 95%	
				Lower limit	Upper limit
Mortality per 1 point increase in variable	Age [years]	1.30	0.38	0.72	2.35
	MELD	1.99	** < 0.001**	1.34	2.98
	CIRS-CI	0.54	0.22	0.32	1.28
	BMI [Kg/m^2^]	2.34	** < 0.001**	1.36	4.05
	SPPB	0.77	0.32	0.46	1.28
	Handgrip [Kg_f_]	0.88	0.45	0.75	0.99
	MNA	0.57	**0.01**	0.36	0.89
	MMSE	1.32	0.51	0.57	3.05
	Poor ADL	3.34	**0.04**	1.08	4.20
Mortality per specified category	Sex M	0.10	**0.01**	0.00	0.27
	Polypharmacy (≥ 5 drugs)	1.60	0.17	0.81	3.35
	Active smoker (yes)	2.06	0.52	0.23	4.51
	Alcohol consumption (yes)	0.16	0.13	0.01	1.70
	Encephalopathy (yes)	0.56	0.81	0.01	2.65
	HCC (yes)	0.13	0.26	0.01	4.38

*p*-values < 0.05 are identified in bold

*HR* Hazard ratio, *MELD* Model for end-stage liver disease, *CTP* Child–turcotte–pugh, *CIRS-CI* Cumulative Illness Rating Scale-Comorbidity Index, *HCC* Hepatocellular carcinoma, *BMI* Body Mass Index, *SPPB* Short physical performance battery, *MNA* Mini nutritional assessment, *MMSE* Mini-mental state examination, *ADL* Activities of daily living

## Discussion

Our study confirmed gender differences in end-stage disease, with high prevalence of HCC in older men, congruent with a significant increase in end-stage liver disease due to alcohol consumption, and higher frequency of ascites and hepatic encephalopathy in women. With regard to the aim of the study, in older people patients with end-stage liver disease, MPI was as accurate as LFI, or even slightly better, in identifying frail patients at higher risk of mortality. High MELD, high BMI, and poor ADL scores were found to be significant risk factors for mortality, while male sex and good nutritional status were protective factors.

Liver transplantation should be considered in all patients with end-stage disease for whom the procedure could improve life expectancy and quality of life [2]. Given the role of frailty in the progression of chronic diseases [8], in recent years it has become an important target for assessment in the selection of patients for liver transplantation. The importance of frailty as a predictor of mortality in patients listed for liver transplantation was first observed by Lai et al. [26] in 2014, and was subsequently confirmed by other authors [27–29]. Previous studies had used Linda Fried's criteria for the definition of frailty, that is, the presence of at least three of involuntary weight loss, reduced muscle strength (handgrip), slowness of walking, low level of physical activity, and fatigability [30]. Over the years, a new score based on this definition was developed specifically for liver patients: the LFI [31]. Although it is better than MELD alone in predicting mortality [13], studies on older adults are still lacking, and, to our knowledge, this is the first study to apply LFI to an exclusively geriatric population. Considering the huge impact that frailty has on older adults, assessment of it is one of the objectives of geriatric medicine [32]. As homeostatic instability due to a frail status involves multiple physiological domains [15], making patients more vulnerable to stressful situations [33], a multidimensional approach to assessing older adults is required. MPI, developed to evaluate frailty status in hospitalized patients [15, 33–35], is one of the most commonly used prognostic tools. Given the accuracy of MPI in stratifying a population according to short- and long-term mortality risk, it has been applied to several cohorts of older people with specific acute and chronic diseases, such as cardiology [36], oncology [37], nephrology [38], gastroenterology [39], and surgery [17]. Based on these premises, we compared the accuracy of LFI and MPI in older adults listed for liver transplantation. Our results confirmed MPI as having good accuracy also in liver transplant patients, with an AUC value comparable to or even slightly better than that of LFI, making it an appropriate tool for use with this population. In our opinion, the added value of MPI lies in it being multidomain. This tool is in fact based on a multidimensional model of frailty, composed not only by subcellular biological and physiopathological mechanisms, but also by clinical consequences and manifestations, i.e., functional deficits, reduced mobility, cognitive impairment, loss of independence in the activities of daily living, and multiple chronic diseases. In this scenario, multimorbidity and polypharmacy are seen both as causes and effects [15]. Because of this, some items included in MPI are actually susceptible to target interventions (e.g., nutritional or functional) that could improve the patient’s prognosis by reducing the burden of risk factors potentially complicating or counterindicating liver transplantation. This makes it a more manageable and useful tool than LFI for use with older adult liver transplant candidates, although further studies with larger samples are needed to confirm this. On the other side, given its 8 domains and 51 items, the calculation of MPI could appear difficult and time costly; for these reasons, Geriatrician is the expert physician who better knows the complexity of multidimensional evaluation and perform it to ordinarily. However, the time needed for the complete compilation of MPI is about 15–25 min, a remarkably short period of time considering the complexity required by the assessment of frailty and the accuracy of this instrument in predicting mortality [40]. Moreover, recently a short form (called Brief-MPI) has been proposed, preserving the multidimensional value of the original form and proving arguably more access to any physician, regardless of his specialty [41].

Finally, with regard to predictors of mortality, the Cox regression model confirmed the role of variables such as high MELD and BMI scores, and loss of functional autonomy as risk factors, and high MNA scores and male sex as protective factors. Our results are in line with other previously reported results [42–45]. Nair et al. suggest that patients with BMI > 35 kg/m^2^ listed for liver transplantation should be encouraged to lose weight to avoid adverse cardiovascular events that could compromise survival over the subsequent 5 years [42]. Furthermore, the severely obese often had high MELD scores, partially explaining the high mortality rates observed in these patients [43]. In this context, malnutrition as measured by the MNA can also reduce the chances of survival, compromising the efficacy of the transplant, and can predispose the patient to post-transplant infectious complications, given also the high impact of immune dysfunction [43]. Regarding the role of sex, recent data published in the USA have reported reduced mortality rates from liver cirrhosis in older women compared with men [46]. In line with our results, Lai et al. reported high mortality rates in women liver transplantation candidates, resulting from the high prevalence of frail participants in their female sample [45]]. LFI and MPI scores did not significantly differ between men and women in our study, but rather they appeared to be related to the higher number of portal hypertension comorbidities in women.

The main limitation of this study is its being a single-center study; due to our strict inclusion criteria, the number of participants limited the statistical power of analyses. Moreover, the decision-making process for transplantation was still based on a multidisciplinary and multidimensional approach, not always resorting to standardized tools. On the other side, the strengths are the selection of an exclusively older people patient cohort, multidimensional assessment using multiple tests, and the inclusion of the MPI as a frailty assessment tool.

## Conclusion

In older adults patients listed for liver transplantation, MPI is as good a prognostic tool as LFI for predicting mortality; high MELD and BMI scores and the loss of functional autonomy were associated with a high risk of mortality, while good nutritional status and male sex emerged as protective factors. Further investigations are needed to investigate the effectiveness of MPI as a predictor of pre- and post-transplant mortality, not only in the older people population, but also in the general population.

## Data Availability

All data generated or analyzed during this study are included in this article. Further enquiries can be directed to the corresponding author.
